# Effect of Antioxidants on High-Temperature Stability of Renewable Bio-Oils Revealed by an Innovative Method for the Determination of Kinetic Parameters of Oxidative Reactions

**DOI:** 10.3390/antiox9050399

**Published:** 2020-05-08

**Authors:** Fabio Mollica, Marco Lucarini, Cinzia Passerini, Claudio Carati, Silvia Pavoni, Lucia Bonoldi, Riccardo Amorati

**Affiliations:** 1 Department of Chemistry “G. Ciamician”, University of Bologna, Via S. Giacomo 11, 40126 Bologna, Italy; fabio.mollica3@unibo.it (F.M.); marco.lucarini@unibo.it (M.L.); 2 Research and Technological Innovation Department, Eni SpA, Via F. Maritano 26, I-20097 San Donato Milanese, Italy; cinzia.passerini@eni.com (C.P.); Claudio.carati@eni.com (C.C.); Silvia.pavoni@eni.com (S.P.); lucia.bonoldi@eni.com (L.B.)

**Keywords:** oxidative stability, biodiesel, induction period, propyl gallate, oil mixtures, antioxidant, oxygen consumption, kinetics, radicals

## Abstract

Bio-oils employed for various industrial purposes, such as biodiesel production, undergo extensive oxidation and degradation during transformation processes. Therefore, it is extremely important to predict their stability at high temperature. We report herein a new procedure based on the optically detected profile of headspace O_2_ concentration during isotherms at 130 °C for evaluating the oxidation kinetic parameters of several bio-oil feedstocks. The slope of O_2_ consumption and the induction period duration were related to the oil characteristics (molecular structure, acidity, and presence of intrinsic antioxidants or metals). The increase of the induction time caused by a standardized propyl gallate addition yielded a semiquantitative value of radical generation rate. Investigated oils included used cooking oils; mono-, di-, and triglycerides from natural sources; free fatty acids; transesterified oils; and their blends. With respect to other methods, this characterization presents the advantage of disentangling and evaluating the role of both fatty acids composition and naturally occurring antioxidants, and allows the development of rational strategies for antioxidant protection of oils and of their blends.

## 1. Introduction

Replacing fossil-derived energy sources with renewable feedstock is important to cope with the gradual depletion of nonrenewable fossil fuels, pollution, and excessive CO_2_ emissions [1]. The conversion of waste and used cooking vegetable oils to renewable diesel is sought as a profitable source of biofuels, because it is less energy demanding than processes involving different feedstock, for instance lignocellulosic biomass [2]. First-generation biofuels, mainly consisting of fatty acids methyl esters, has specific disadvantages caused by their high oxygen content, such as a poor calorific value, low storage stability, high viscosity, and density. This prompted the development of a second generation of biofuels, based on the simultaneous deoxygenation and hydrogenation of first-generation biodiesel [3]. Up-conversion, however, requires the storage and manipulation of feedstocks at relatively high temperatures, which is a well-known condition that increases degradation processes [4]. In fact, degradation of natural oils at high temperature is a problem occurring in many technological applications, ranging from food deep-frying [5,6] to biodiesel processing [7]. Thermal stress causes oxidation and polymerization of oils, with the formation of volatile, non-volatile, and polymeric compounds that affect the oil chemical-physical characteristics and lead to the formation of fouling and solid deposits. The extent of this process depends on many parameters, the most important being the oil composition, temperature, and accessibility of oxygen [8]. When O_2_ is present in the system, oxidation is the most relevant oil degradation mechanism, consisting of a free-radical mediated autoxidation, which leads to the formation of hydroperoxides and other oxygenated compounds. In turn, hydroperoxides undergo homolytic cleavage and increase the decomposition by generating new radicals [9]. The extent of autoxidation is dependent on oil composition, as the susceptibility of fatty acids toward oxidation increases in the order: saturated < monounsaturated < polyunsaturated [9]. It is further promoted by the presence of metals [9]. Oil stability is instead increased by the presence of antioxidants which may be naturally present in the oil or can be added on purpose. Natural tocopherols, which are commonly present in seed oils, have poor performances compared to synthetic antioxidants such as BHT, gallate esters, and butylated hydroquinones [7]. In addition, unsaponifiable oil components, including phytosterols and oryzanol, have been found to increase oil stability presumably by a mechanism not related to radical trapping [8].

**Scheme 1:**
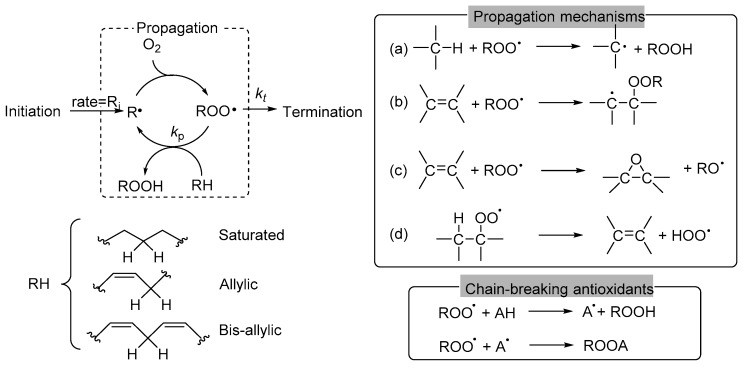
Mechanism of autoxidation of natural fatty acids. On the top right, the main mechanism of propagation by peroxyl radicals: (**a**) H-atom transfer; (**b**) addition; (**c**) addition–fragmentation; and (**d**) generation of a hydroperoxyl radical. In the bottom right is the general mechanism of action of chain breaking antioxidants.

The chemical mechanism of oil autoxidation is a radical chain, as described in Scheme 1 [10]. Initiation processes generate alkyl radicals (R^•^) that react with O_2_ at a diffusion-controlled rate yielding alkyl hydroperoxides (ROO^•^). Initiation occurs through various processes including the decomposition of traces of hydroperoxides, possibly catalyzed by transition metals, or by the direct reaction of activated C-H bonds with O_2_ [11]. Peroxyl radicals then propagate the oxidative chain by different pathways, as summarized in Scheme 1 [12,13,14].

Chain breaking antioxidants such as butylated hydroxytoluene (BHT) or propyl gallate (PG) act by trapping peroxyl radicals through the reactions depicted in Scheme 1. Following this general oxidation scheme, the duration of the inhibition period of an antioxidant (*τ*) is proportional to its concentration and inversely proportional to the initiation rate (*τ*∝[AH]/*R*_i_) [15,16].

The oxidation stability can be measured by different parameters, such as iodine value, anisidine value, peroxide value, and Oxidative Stability Index (OSI) (also named Induction Period (IP)). The iodine value is related to the concentration of double bonds in the pristine oil, which is more oxidizable if it has a higher unsaturation level, while peroxide and anisidine values are related to the oxidation degree [17]. Generally speaking, oxidation proceeds through the formation of primary and secondary oxidation products, i.e. first peroxide and conjugated dienes, and then carbonyls and acids [18]. The peroxide value is related to the concentration of hydroperoxides and therefore highlights the dimension of the first oxidation step, while the anisidine value (AOCS Official Method Cd 18-90) is related to the concentration of aldehydes and ketones, and accounts for the dimension of the secondary oxidation process [18]. These analytical methods for the quantitative determination of specific products are very accurate; however, they are experimentally demanding, and may present the limit of partial information. In addition, they are focused on the oxidation level, but not directly on the kinetics of the oxidation process, that is instead investigated by OSI method. The OSI method (AOCS Cd12b-92) is performed by passing purified air through a sample that is kept under high temperature (70–110 °C), promoting a rapid degradation of the triglycerides with formation of volatile organic acids, conveyed by the air stream into a conductivity cell full of water where the acids are solubilized. These acids, once dissolved in the water solution, dissociate into ions, thus changing the conductivity of the water. The time requested to induce the rapid rise in conductivity (hours) is the OSI time [17,19]. This method is largely applied for the characterization of oxidation stability of oils and fats, and particularly in the case of biodiesel of various origins in both academic studies and industrial applications [20,21]. The OSI method measures the duration of the inhibited period, which, as discussed above, depends both on the type and concentration of intrinsic antioxidants and on the rate of radical initiation, more related to the oil bulk composition.

Having a separate evaluation of factors underlying oil autoxidation (that are initiation, propagation, and inhibition) would be of fundamental importance to understand and control this unwanted process. For this reason, to improve our knowledge of oil oxidative reactions at high temperature, we studied the full profile of O_2_ uptake of several samples of oils during isotherms at 130 °C by an optical O_2_ probe. The study of O_2_ uptake is among the best methods to follow autoxidation reactions, because there is little interference with other processes [12,13,14,15,16]. For instance, hydroperoxides and aldehydes are known to be degraded by radical and non-radical reactions, therefore their level is not always related to the substrate oxidation [7,9]. In addition to the duration of the inhibited period, this experimental setup allowed measuring the O_2_ consumption rates before and after the onset of the fast oxidation regime, i.e. three independent parameters describing oil oxidation, and shedding some light on the separate factors impacting on it. We also performed these kinetic measurements in the presence of a fixed amount of the antioxidant propyl gallate, as an expedient to have a semiquantitative evaluation of the magnitude of the initiation rate *R*_i_. Our results show that the oil composition is the most important variable influencing both the oxidation rate and the duration of antioxidants and allow us to make a prediction about the resistance toward oxidation of oils blends.

## 2. Materials and Methods

### 2.1. Materials

HPLC grade 1,2-dichlorobenzene and PG (propyl gallate) (>98%) were from Sigma-Aldrich (Milan, Italy) and 1-octanol (99%) was from Alfa Aesar (Erlenbachweg, Germany). They were used as received. Oils were provided by Eni S.p.A. Research Center (San Donato Milanese, Italy) and were stored in a refrigerator in the dark. Peroxide levels were ≤23.5 mEq O_2_/kg and are reported in the Supplementary Materials (Table S1)). Sampling of viscous or solid oils was done after stirring at 60 °C for a short time.

### 2.2. Oil Characterization

Average composition was analyzed by NMR. The samples were diluted 30% *v*/*v* in deuterated chloroform (CDCl_3_) and ^1^H and ^13^C NMR spectra recorded on a 500 MHz Varian V500 spectrometer (Varian Inc., Palo Alto, CA, USA) [22]. Iron (and other elemental content) was determined by ICP-OES (ICAP 6500 DV Thermo Scientific, Waltham, MA, USA), following the UOP 389 reference method, consisting of sample preparation by ashing and acidic digestion of ashes. Quantitative determinations were duplicated [23]. The full elemental content is reported in the Supplementary Materials. 

The acid value was measured following ASTM D664, Method B for biodiesel and blends using the automated titrator model Titrando 905 (Metrohm, Herisau, Switzerland) equipped with the pH sensing electrode model Solvotrode easyClean (Metrohm, Herisau, Switzerland) The results are expressed as mg KOH required to neutralize 1.0 g of the biodiesel sample [24].

### 2.3. Oxidative Stability Index

Oxidative stability indices (OSI) were expressed as induction periods in hours and determined according to the EN15751 standard on a Rancimat apparatus (Metrohm, Herisau, Switzerland) [25,26,27].

### 2.4. Measure of Oxygen Consumption

**Figure 1:**
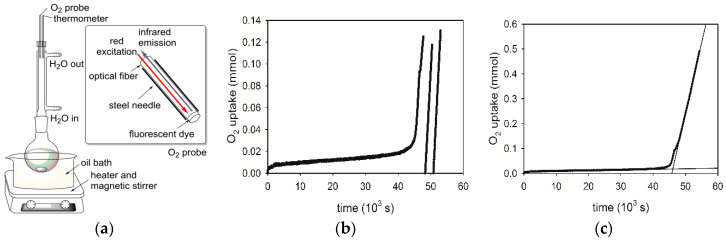
(**a**) Apparatus for measuring oxygen consumption and scheme of the optical O_2_ probe. (**b**,**c**) Experimental oxygen uptake of jojoba oil (BIO86) at 130 °C: (**b**) results from three open–close cycles; and (**c**) plot obtained by connecting the traces of plot (**b**).

Oxygen consumption was measured in a 10 mL round bottom flask surmounted by a short glass condenser, over which the optical oxygen meter and the thermometer were introduced through a silicone rubber septum (see Figure 1a).

The operating principle is based on luminescence quenching of a sensor dye. The dye is excited with red light, and the properties of the resulting luminescence are measured in the near infrared. The presence of molecular oxygen quenches the luminescence, changing its intensity and lifetime fully reversibly. The probe provides the direct measure of O_2_ concentration and shows virtually no interferences to other gases. The probe was a Robust Oxygen Probe manufactured by Pyroscience GmbH (Aachen, Germany), coupled to a FireStingO_2_ control unit having a thermometer probe for continuous temperature correction (response time <7 s, accuracy 0.2%, resolution 0.05% at 20% O_2_, temperature range from 0 to 50 °C, and one-point calibration under ambient air). The probe provides the direct measure of O_2_ concentration and was calibrated under air by following the manufacturer’s instructions. The glass condenser maintains the probe tip at about 30 °C and this equipment is suited for measuring O_2_ uptake up to 180 °C. Samples consisted of 1 mL of oil dissolved in 7 mL 1,2-dichlorobenzene (boiling point = 180 °C) vigorously stirred by an olive-shaped stir bar and heated to the required temperature by a silicone oil bath. The dilution degree was optimized by preliminary experiments to avoid that O_2_ transfer from air to the sample is rate limiting. When approximately 75% O_2_ was consumed, the O_2_ uptake rate gradually slows down. To measure the oxygen consumption rate in the fast regime with the maximum accuracy, after the complete consumption of O_2_, fresh air was introduced, the apparatus was sealed again, and the acquisition of kinetic data continued (Figure 1b). This procedure was repeated until the maximum O_2_ consumption rate was constant. The traces of O_2_ consumption measured at the initial part of the reaction (that is, excluding the parts in which the reaction was slowed down by an insufficient O_2_ concentration) were joined in a unique plot, as shown in Figure 1c, and the slope evaluated. The procedure of joining the O_2_ consumption plots after discarding the final part introduces uncertainty regarding the duration of the inhibited period only if the data break occurs before the end of the inhibition period (*τ*). In our experiments, the inhibited period was contained in the first experiment, and thus the subsequent opening–closing did not modify *τ* length. The O_2_ concentration was collected every 10 s, and the readings were recorded by a computer by using the probe manufacturer acquisition software. The measure had a dead time of about 1000 s required for thermal equilibration of the sample and for O_2_ diffusion inside the condenser.

To have selective information on the initiation rate *R*_i_, the kinetics were repeated on the fresh oils added with PG dissolved in 1-octanol to yield a final concentration of 500 ppm.

All the oxidation reactions were repeated twice and the errors, defined as the distance between the mean and the experimental values, were below 10%.

Multiple linear regression analysis was performed by SigmaPlot software (version 11.0, Systat Software Inc., San Jose, CA, USA).

## 3. Results

### 3.1. Analysis of the O_2_ Consumption Plots

**Figure 2:**
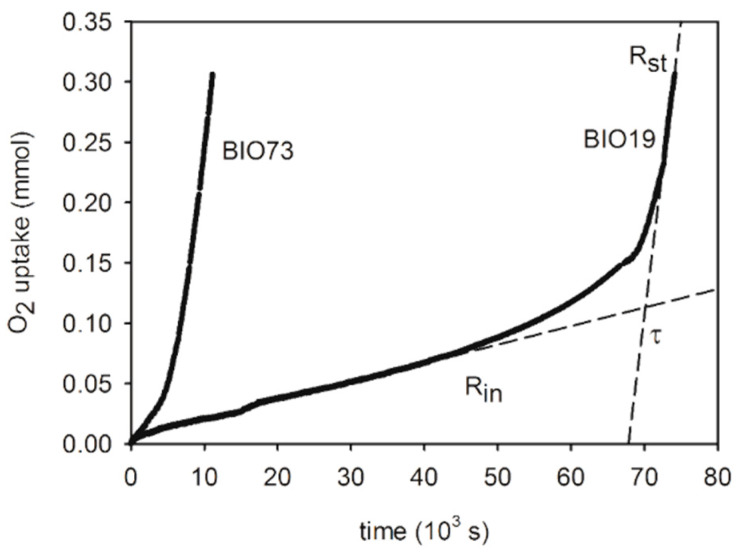
O_2_ uptake measured during the oxidation at 130 °C of raw (BIO19) and purified (BIO73) palm oil.

The oils considered in the present study included triglycerides; mixtures of mono-, di-, and triglycerides in different proportions; free fatty acids; transesterified oils; waste oils; and oils deriving from wood processing, to reflect the diversity of bio-based matrixes used to produce biodiesel (see Table 1). Based on the shapes of the oxygen consumption plots, measured in the headspace of a stirred reaction vessel at 130 °C, oils were divided into three families: (A) linear O_2_ consumption from the beginning of the experiment (Type A oils); (B) initial slow O_2_ consumption (Type B oils); and (C) initial fast O_2_ consumption (Type C oils). The symbols used to indicate these periods are: *τ* = duration of the induction period; *R_in_* = initial rate; and *R_st_* = steady rate (see Figure 2). The results of O_2_ consumption experiments are reported in Table 1.

### 3.2. Inhibition Period (Type B oils)

**Figure 3:**
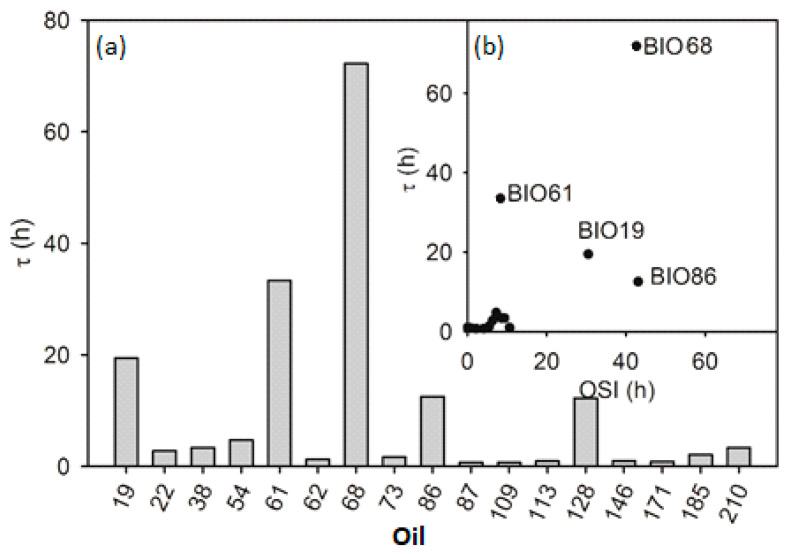
(**a**) Induction period of Type B oils. (**b**) The inset reports the relationship between OSI values and *τ*, showing that t is in general smaller than OSI, with the exception of BIO61 and BIO68.

Oils belonging to Type B showed an induction period before the onset of fast O_2_ consumption, which can be attributed to the presence of intrinsic antioxidants in the oils. For example, the two samples BIO19 and BIO73 are constituted by raw and purified palm oil, respectively. In Figure 2, it is shown that the inhibition period of BIO19 is much longer than that of BIO73, despite the former contains higher levels of iron, a well-known oxidation catalyst. We explain this with the removal of endogenous antioxidants during the purification treatment. Interestingly, the slopes of O_2_ uptake after the end of the induction time *R*_st_ of the two oils are almost coincident, indicating that *R*_st_ reflects the composition of triglycerides while other minor differences (see Table 1) are not relevant. The length of the induction periods of Type B oils are reported in Table 1 and Figure 3a. The length of the induction period (*τ*) was related to the Oil Stability Index (OSI), which represents the induction time at 110 °C measured by the Rancimat apparatus.

If comparing the two set of values (Figure 3b), it can be noted that *τ* is in general shorter than OSI, reasonably as the effect of the higher temperature that leads to greater production of radicals and hence to faster consumption of the antioxidants. In most cases, the two parameters show similar stability ranking of the oils. Some exceptions in the general agreement could be due to the different experimental setup, or to specific chemical effects involving the chain-carrying radicals.

### 3.3. Steady Oxygen Consumption (R_st_) in Type A–C Oils

**Figure 4:**
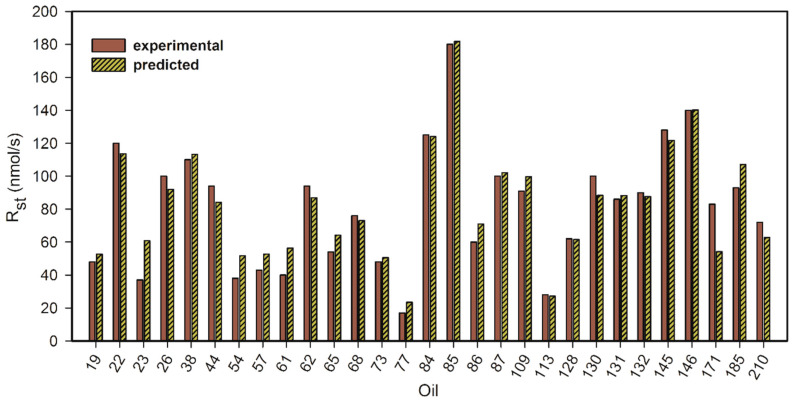
Overview of experimental *R*_st_ of oils at 130 °C and comparison with the values predicted by the multiple linear regression shown in Equation (2).

A steady oxygen consumption is shown by Type A oils soon after the beginning of the oxidation, while in the case of Type B and C oils, it is reached after an initial inhibited or accelerated period. The steady oxygen consumption measured for all oils, reported in Figure 4, showed a marked variation among the oils, ranging from 17 nmol/s of a fully saturated oil (BIO77) to 180 nmol/s of linseed oil (BIO85). To understand in detail the determining factors of oil oxidation, *R*_st_ dependence on oil characteristics was analyzed by multiple linear regression. This procedure showed that the main determinant of *R*_st_ was the percent concentration of mono (MO)-, di (DI)-, and tri (TRI)-unsaturated fatty acids (Equation (1); *r*^2^ = 0.78); however, the best results were obtained when also the oil acidity (H^+^, expressed as mg KOH/g) was considered, as shown in Equation (2) (*r*^2^ = 0.92).

*R*_st_ (nmol/s) = 20.1 + (0.570 MO%) + (1.54 DI%) + (2.65 TRI%) (1)

*R*_st_ (nmol/s) = 9.82 + (0.634 MO%) + (1.63 DI%) + (2.65 TRI%) + (0.154 H^+^) (2)

Inclusion of the iron content or peroxide value (see Table S1) caused a worsening of the regression coefficient. While the dependence of *R*_st_ on oil unsaturation is not unexpected, the role of acid is less clear and will be the object of further studies.

### 3.4. Standard Addition of Propyl Gallate

**Figure 5:**
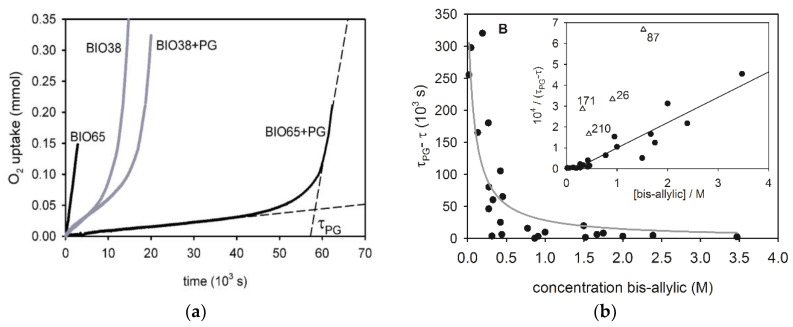
(**a**) Effect of propyl gallate (PG, 500 ppm) on the oxidation of two different oils; and (**b**) relationship between the PG effect (*τ*_PG_-*τ*) and oil composition. Oils showing no PG effect because of high iron or acidity contents were not included. The inset shows the linear relationship between 1/(*τ*_PG_-*τ*) and the concentration of bis-allylic groups, white triangles indicate outliers (see text).

To get further information on oil behavior, we performed the oil oxidation with the addition of 500 mg/L of propyl gallate (PG), a widely used antioxidant for oil stabilization, to every sample. The aim of these experiments was to classify oils by measuring the duration of the inhibition period given by a constant amount of the same antioxidant (PG), while the activity of naturally occurring antioxidants is in general different. The effect of PG (*τ*_PG_-*τ*) was obtained from the difference between the inhibition in the presence (*τ*_PG_) and in the absence of PG (*τ*), as shown in Figure 5a.

It can be noted that the slope of O_2_ uptake at the end of the induction period (i.e., *R*_st_) with or without PG are essentially the same, confirming that, when the concentration of antioxidants expires, the oxidation process of the bulk oil is the same (for instance, *R*_st_ was 54 and 50 nmol/s for BIO65 and BIO65+PG, respectively). The results collected in Table 1 clearly show that the effect of PG is nearly zero in oils having a high iron content (see, for instance, BIO57 and BIO130–132), or high acidity (see BIO145). If comparing for instance the palm oils samples (BIO19, BIO73, and BIO185), having comparable fatty acid composition, the first two showed a high PG effect (80 × 10^3^ s and 46 × 10^3^ s, respectively), whereas BIO185, having a large acidity and iron content, displayed a relatively small PG effect (6.5 × 10^3^ s). The effect of PG on oil oxidation was then analyzed by various regression methods, excluding those oils having high iron and acidity content, marked in bold in Table 1. The best fitting, reported in Equation (3), is an inverse dependence on the concentration of bis-allylic groups in the sample (Figure 5b), while other parameters such as the peroxide or acidity levels, had no significant role.

*τ*_PG_-*τ* = 3.0 × 10^4^/(0.080 + [bis-allylic]) (3)

This agrees with previous reports showing that the effect of antioxidants is low in oils having a high unsaturation degree [23]. If plotting the same data as 1/*τ*_PG_-*τ*) vs. [bis-allylic], a fairly straight relationship (see Equation (4), *r*^2^ = 0.87) is found with a few outliers, reasonably due to the interaction of PG with impurities [28,29,30]. This plot is shown in the inset of Figure 5b and the outliers are indicated by a triangle.

1/(*τ*_PG_-*τ*) = 1.2 × 10^−4^ × [bis-allylic] – 2.2 × 10^−5^ (4)

The physical meaning of these relationships is explained in the Section 4 on the basis of the kinetic equations describing oil oxidation.

### 3.5. Initial Fast O_2_ Consumption (Type C Oils)

In the case of two oils, BIO69 and BIO77, the oil oxidation started with a high rate of O_2_ uptake for a short time, which then decreased to reach a constant value. By inspecting the results reported in Table 1, the final O_2_ consumption rate (i.e., *R*_st_) for Type C oils were the lowest among all oils. This can be explained by considering that BIO69 and BIO77 are almost completely composed of saturated fats, so that *R*_st_ is low, while the presence of traces of more oxidizable material is responsible for the initial oxidation burst. The addition of PG caused a slow-down of O_2_ consumption in the case of BIO77 (from 17 to 10 nmol/s) with no inhibition period, probably because the low R_i_ of this oil renders *τ*_PG_ exceedingly long. In the case of BIO69, PG had no effect presumably because the high iron level found in this oil.

### 3.6. Oil Mixtures

**Figure 6:**
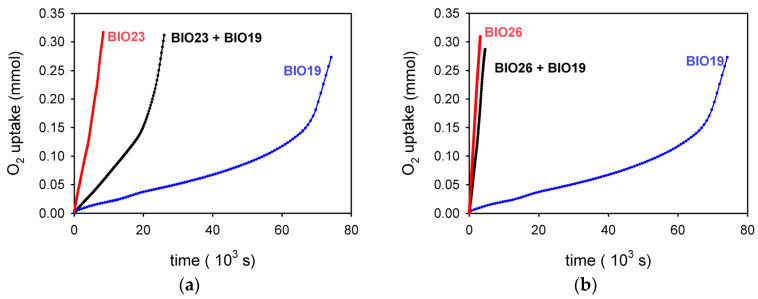
O_2_ uptake measured during the oxidation at 130 °C of oil mixtures. (**a**) BIO 23 and BIO19, (**b**) BIO26 and BIO19.

The behavior of mixtures of oils having different inhibition periods was then investigated, as oils blends have been proposed as a strategy to improve their oxidative stability [31,32]. Palm oil (BIO19) or jojoba oil (BIO86), chosen because of their long inhibition period, were mixed in equal amounts to BIO23, BIO26, or BIO44, having almost no induction period (see Figure 6).

The results, reported in Table 2 show that the addition of BIO19 and BIO86 could improve the stability only of BIO23, while in other cases the induction period of the mixture was almost the same as that of the most unstable component. This result can be understood in light of the effect of PG (*τ*_PG_-*τ*) on these oils. An oil can be characterized by a short induction period for two different reasons: (i) high *R*_i_ (corresponding to a low PG effect) that causes the quick consumption of intrinsic antioxidants (if present); and (ii) lack of intrinsic antioxidants.

The present results suggest that only oils that lack an induction period and have a low *R*_i_ (high *τ*_PG_-*τ*) such as BIO23, can be effectively stabilized by mixing them to stable oils. Instead, oils with high *R*_i_ (low *τ*_PG_-*τ*) quickly consume the antioxidants provided by the stable oil. In the cases of the oils investigated, the effect of reducing the bis-allylic group concentration by the addition of oils rich in saturated or monounsaturated fatty acids plays a minor role, differently from what observed in low temperature stability experiments [33]. These results show that in selected cases the mixture of two oils with different stability can improve the stability of the less stable one.

## 4. Discussion

The autoxidation of a lipid substrate (indicated herein as RH) in the absence of antioxidants is described by Reactions (5)–(8), where *k_p_* and *k_t_* are the propagation and termination *rate constants*, respectively, and *R_i_* is the initiation *rate* [10,15].

(5) $$Initiator\overset{R_{i}\left(Ms^{-1}\right)}{\to }R•$$

(6) $$R•+O_{2}\overset{k\approx 10^{9}(M^{-1}s^{-1})}{\to }ROO•$$

(7) $$ROO•+RH\overset{k_{p}\left(M^{-1}s^{-1}\right)}{\to }ROOH+R•$$

(8) $$2\;ROO•\;\overset{k_{t}\left(M^{-1}s^{-1}\right)}{\to }non-radical\;products$$

(9) $$ROO•+AH\;\overset{k_{inh}\left(M^{-1}s^{-1}\right)}{\to }ROOH+A•$$

(10) $$A•+ROO•\;\overset{k\approx 10^{8}(M^{-1}s^{-1})}{\to }ROOA$$

This set of reactions is valid if O_2_ concentration is large enough to transform all carbon-centered radicals (R^•^) into alkylperoxyl ones (ROO^•^), so that other reactions of R^•^ can be neglected, and if generation of new radical due to hydroperoxides (ROOH) homolysis can be excluded. This is the case in our experimental conditions. In fact, if ROOH generated radicals, the rate of O_2_ consumption would increase with time [10,15], which we did not experimentally observe. We found instead a constant O_2_ consumption rate (*R*_st_) in absence of antioxidants, indicating that ROOH do not participate in the initiation process, and that the initiation rate (*R*_i_) is constant throughout the experiment. This was also true in the case of large iron concentration. Therefore, peroxide dissociation can be ruled out, the rate of initiation *R*_i_ does not change during the reaction, and the rate of oxygen consumption during the autoxidation of a generic oxidizable substrate RH in the absence of antioxidants (*R*_st_) is given by Equation (11), which can be obtained by the mathematical solution of Equations (5)–(8) by applying the steady-state approximation to all radical species (see Supplementary Material), and by assuming that RH and O_2_ consumption is negligible [34].

(11) $$\mathrm{steady}\mathrm{state},\mathrm{slope}\;\;\;-\frac{d\left[O_{2}\right]}{dt}=R_{st}=\frac{k_{p}}{\sqrt{2k_{t}}}\left[RH\right]\sqrt{R_{i}}+R_{i}$$

The values of *k*_p_ and *k*_t_ are determined by the chemical structure of the fatty acids. For instance, at 30 °C, *k_p_* (for each H, M^‒1^s^‒1^) is 0.004, 0.22, and 31 for saturated, allylic, and bis-allylic functional groups, respectively, while *k*_t_ is approximately constant (≈10^7^ M^−1^s^−1^) [14].

In the presence of an antioxidant (AH), ROO^•^ radicals are terminated by Reactions (9) and (10). Reaction (9) is the rate limiting step of the process, therefore *k*_inh_ is the rate constant that describes the efficacy of an antioxidant. Reaction (10) accounts for the fate of the A^•^ radical and establishes the number of radicals trapped by each AH molecule (i.e., the stoichiometric coefficient of the antioxidant, *n*). If A^•^ is stable enough to react with a second ROO^•^ (as shown in Reaction (10)), the value of *n* is 2. If A^•^ decays by following different pathways (such as dimerization or reaction with O_2_), smaller *n* values are observed. In the reasonable assumption that, in the presence of an antioxidant, ROO^•^ radicals disappear only by Reactions (9) and (10), the oxygen consumption rate (*R*_in_) at the *beginning* of the induction period is described by Equation (12), where *k_inh_* is the rate constant for the reaction of peroxyl radicals with the antioxidant and *n* is the number of radicals trapped by each antioxidant molecule [10,26]. Equation (12) is derived by applying the steady-state approximation to all radical species, and by assuming that RH and O_2_ consumption is negligible (see Supplementary Material). The duration of the inhibition period (*τ*) is proportional to the concentration of the antioxidant and inversely proportional to the initiation rate (Equation (13)) [10,15,35].

(12) $$\mathrm{inhibition}\;\mathrm{period},\;\mathrm{slope}\;\;\;-\frac{d\left[O_{2}\right]}{dt}=R_{in}=\frac{k_{p}\left[RH\right]R_{i}}{n\;k_{inh}\left[AH\right]}+R_{i}$$

(13) $$\mathrm{inhibition}\;\mathrm{period},\;\mathrm{duration}\;\;\;\tau =\frac{n\left[AH\right]}{R_{i}}$$

These equations provide some useful indications that can help to rationalize the results obtained.

- Equation (11) describes the oxygen consumption in the steady state regime (*R*_st_), after the conclusion of the inhibition period or in case the oil does not contain antioxidants. In these conditions, the rate of oil oxidation depends on the concentration of the oxidizable groups, the facility of oxidation of fatty acids (i.e., on *k_p_/√2k_t_*), and *R_i_.* As we found linear O_2_ consumption, we deduced that in our system the initiation rate (*R_i_*) is constant. The effect of oil acidity may influence *k*_p_, *k*_t_ or both.
- The presence of an inhibition period is clear experimental evidence of the presence of a naturally occurring antioxidant in the oil. It finishes when the antioxidant is consumed. The inhibition period is characterized by its duration and by the slope (−d[O_2_]/dt) that is associated to the effectiveness of the antioxidant in the inhibition of the O_2_ consumption. The slope and duration of oxygen consumption during the inhibition period are described by Equations (12) and (13). From Equation (12), −d[O_2_]/dt = *R*_in_ depends on the concentration of the oxidizable groups, the nature of the oil (*k_p_*), the antioxidant (*n* and *k_inh_*) and the rate of initiation (*R_i_*).
- The duration of the inhibited period (Equation (13)) depends not only on the type and concentration of antioxidants present in the oil but also it is inversely proportional to the rate of initiation *R*_i_. For this reason, the addition of a known fixed quantity of a specific antioxidant provides an estimate of the *R*_i_ value of the oil, as we did with the addition of propyl gallate (PG). As the *n* value for PG at 130 °C is not known, we could obtain only the *R*_i_ / *n* ratio. Interestingly, when excluding oils with high acidity, iron content, and a few other outliers in which PG exerted little or no inhibiting activity, there is a good inverse correlation between *τ*_PG_-*τ* and the concentration of bis-allylic groups in the oils with y-intercept close to zero. Therefore, it can be inferred that *R*_i_ is directly proportional to the concentration of bis-allylic groups. This result confirms the hypothesis that the initiation of oil oxidation at 130 °C is mainly due to the direct reaction of activated C-H bonds with O_2_ [11].

## 5. Conclusions

Herein, we report the study of the O_2_ uptake profile of several samples of oils during isotherms at 130 °C, measured by an optical O_2_ probe. The procedure is suitable for pure and diluted oils and for a wide range of temperatures up to 180 °C. The analysis of the curves obtained by this experimental setup allowed measuring various parameters describing oil oxidation, and thus to disentangle the role of fatty acids composition from that of antioxidants or other minor compounds. The addition of a fixed concentration of the antioxidant propyl gallate provided a semiquantitative estimate of the rate of radical generation. This latter parameter depends only on the fatty acid composition and is important to predict the efficacy of the antioxidants on each oil, as well as to predict the resistance to oxidation of oil mixtures. Using this information, it is possible to rationally develop new antioxidants and oil mixtures having increased resistance toward oxidation [36,37,38]. Future work will be aimed at comparing different antioxidants and finding a quantitative measure of the initiation rate. We believe that the oximetric method presented herein can complement the methods aimed at studying lipid peroxidation [38]. The results can be useful for the food and pharmaceutical industry to cope with the rancidity of highly unsaturated natural oil (such as those containing valuable ω3 fatty acids) [38], and for the biodiesel production to preserve feedstock from deterioration [1,2,3,4].

## Figures and Tables

**Table 1:** Oil composition, iron content, acidity, oxidation stability index and parameters for oil oxidation at 130 °C: *τ* = duration of the induction time; *R*_in_ = initial O_2_ consumption rate; *R*_st_ = steady O_2_ consumption rate; *τ*-*τ*_PG_ = propyl gallate effect on induction time.

Oil	Description ^[a]^	*R*_in_ nmol/s	*τ* 10^3^ s	*R*_st_ nmol/s	*τ*-*τ*_PG_ 10^3^ s	Fe ppm	H^+^ mg KOH/g	OSI ^[b]^ h	SA ^[c]^	MO ^[c]^	DI ^[c]^ (TRI) ^[c]^
**Type A**											
BIO26	Refined Used Cooking Oil (T)	0	<1	100	3.0	3	8.55	1.00	19.05	52.72	27.10 (1.13)
BIO44	Used Cooking Oil (T)	0	<1	94	15.5	4.3	8.62	0	26.30	50.60	21.20 (1.90)
BIO57	Oil Distillation pitch (T)	0	<1	38	0	**1472**	**33.79**	0.70	60.05	35.86	0.68 (3.41)
BIO65	Vegetable oil fraction (T)	0	<1	54	60	3.7	31.07	0.83	42.59	46.82	10.59 (0)
BIO84	Safflawer oil (T)	0	<1	120	3.2	<0.5	0.96	2.01	18.30	17.16	64.54 (0)
BIO85	Linseed oil (T)	0	<1	180	2.2	<0.5	1.20	0.10	14.00	22.00	16.00 (48)
BIO130	Tall oil (1) (A) ^[d]^	0	<1	100	0	**31.3**	**159.63**	2.77	52.40	27.80	16.90 (2.90)
BIO131	Tall oil (2) (A) ^[d]^	0	<1	86	0	**67.9**	**155.14**	0.10	51.10	28.90	17.40 (2.60)
BIO132	Tall oil (3) (A) ^[d]^	0	<1	90	0	**35.01**	**155.43**	0.10	52.20	28.00	17.10 (2.70)
BIO145	Tall oil fatty acid (A)	0	<1	128	0	0.6	**193.27**	0.10	30.60	30.94	36.10 (2.36)
**Type B**											
BIO19	Palm oil (T)	1,5	70	48	80	5.4	8.48	30.50	55.06	36.22	8.56 (0.16)
BIO22	Soybean oil (T)	8.9	10.0	120	8	0.5	2.24	6.38	25.17	21.76	49.70 (3.36)
BIO23	Fractioned Seed rape oil (M,D,T)	36	5.5	49	65	9.1	1.39	0.10	48.56	37.49	13.24 (0.70)
BIO38	Corn oil (T)	6.0	12.0	110	6.0	<0.5	0.11	9.40	19.65	27.20	52.50 (0.65)
BIO54	Animal fat (T)	2.2	17.0	38	320	1.1	8.62	7.28	51.63	42.63	5.22 (0.52)
BIO61	Empty fruit bunch (T)	0.88	120	40	180	7.8	33.76	9.12	54.32	37.12	8.44 (0.12)
BIO62	Carinata oil (T)	11	4,5	94	9,5	0.8	0.10	5.53	33.00	40.50	20.75 (5.75)
BIO68	Castor oil (D,T)	0.28	260	76	165	2.1	0.32	44.69	8.17	87.58	4.25 (0)
BIO73	RBD Palm oil (T)	8.25	6	48	46	<0.5	0.21	na	56.8	34.4	8.8 (0)
BIO86	Jojoba Oil	0.24	45	60	255	<0.5	0.47	43.06	5.81	93.55	0.64 (0)
BIO87	Cotton Oil (T)	21	2.5	100	1.5	<0.5	0.10	4.18	31.70	19.25	49.05 (0)
BIO109	Canapa Oil (T)	18.0	2.5	90	19.5	2.4	27.61	2.20	44.20	14.10	34.90 (6.70)
BIO113	Palm kernel oil (T)	5.4	3.5	27	297.5	1	3.94	10.68	87.59	10.93	1.47 (0)
BIO128	Animal fat (ME)	23.0	44	60	25	<0.5	3.12	na	40.2	46.40	11.80 (1.70)
BIO146	Tobacco oil (T)	23	3.2	140	4.6	1.36	7.72	0.10	12.72	11.57	74.34 (1.36)
BIO171	Fatty acids (ME)	36	2.5	85	3.5	4.21	4.96	0.80	53.70	36.10	10.20 (0)
BIO185	Palm oil mill effluent (A)	11	7.5	93	6.5	**126**	**132.02**	0.95	23.89	45.46	30.65 (0)
BIO210	Fatty acids (ME)	8.3	12	72	0.6	0.8	0.18	8.69	43.9	41.8	14.3 (0)
**TYPE C**											
BIO77	C10SE1 (BE) ^[e]^	43	1.0	17	0	3.7	4.57	0.20	92.00	8.00	0 (0)
BIO69	C10SE2 (BE) ^[e]^	54	2.5	18	0	**14.3**	4.57	0.20	92.00	8.00	0 (0)

^[a]^ Legend: T, triglycerides; D, diglycerides; M, monoglycerides; A, free fatty acids; ME, methyl ester; BE, butyl ester. ^[b]^ Oxidative stability index. ^[c]^ SA, saturated; MO, monounsaturated; DI, diunsaturated; TRI, triunsaturated fatty acids, in mol%. ^[d]^ Different batches. ^[e]^ C_10_ saturated fatty acids esters, samples stored in different conditions. Samples with high acidity or iron content (see discussion) are marked in bold.

**Table 2:** Induction period (*τ*) and effect of propyl gallate (*τ*_PG_-*τ*) for selected oils and their blends.

Oil or Mixture	*τ* (10^3^ s)	*τ*_PG_-*τ* (10^3^ s)
BIO19	70	80
BIO23	5.5	65
BIO26	0	3.0
BIO44	0	16
BIO86	45	255
BIO19 + BIO23	23	-
BIO19 + BIO26	0	-
BIO19 + BIO44	4.3	-
BIO86 + BIO23	23	-
BIO86 + BIO44	3.8	-

Samples were prepared by mixing two oils in 1:1 proportion, the overall oil concentration being 12.5% *v/v* in 1,2-dichlorobenzene. No phase separation occurred at 130 °C.

## Supplementary Materials

The following are available online at https://www.mdpi.com/2076-3921/9/5/399/s1, Table S1: Oil characteristics: peroxide value (PV) in mEq O_2_/kg, and full elementary analysis in ppm, derivation of kinetic Equations (11)–(13).
