# Supplementary material for: Effect of Antioxidants on High-Temperature Stability of Renewable Bio-Oils Revealed by an Innovative Method for the Determination of Kinetic Parameters of Oxidative Reactions
Source: Antioxidants (Basel). 2020 May 8;9(5):399. doi: 10.3390/antiox9050399 (PMC7278824; doi:10.3390/antiox9050399)
Supplement: Supplementary file 1 [file antioxidants-09-00399-s001.pdf]

Supplementary Materials

Effect of antioxidants on high-temperature stability of renewable bio-oils revealed by an innovative method for the determination of kinetic parameters of oxidative reactions

Fabio Mollica[1], Marco Lucarini[1], Cinzia Passerini[2], Claudio Carati[2], Silvia Pavoni[2], Lucia Bonoldi,[2] Riccardo Amorati[1]*

[1] Department of Chemistry «G. Ciamician» University of Bologna, via S. Giacomo 11, 40126, Bologna, Italy
[2] Research and Technological Innovation Department, Eni SpA, Via F. Maritano 26, I-20097 San Donato Milanese, Italy

Content: **Page**

Content	Page
**Table s1**. Oil characteristics: peroxide value (PV) in mEq O_2 /Kg, and full elementary analysis in ppm.	2
Derivation of equation for inhibited autoxidation.	4

Table s1. Oil characteristics: peroxide value (PV) in meq. O_2 /Kg, and full elementary analysis in ppm.

	Oil[a]	PV	Al	Ca	K	Mg	Na	Zn	Sn	P
**Type A**										
BIO26	Refined Used Cooking Oil (T)	14.8	<0.5	1.5	12.8	1.0	13.5	0.8	<0.5	3.8
BIO44	Used Cooking Oil (T)	8.1	<0.5	3.5	25.6	2.3	39.9	0.7	<0.5	14.2
BIO57	Oil Distillation pitch (T)	na	36.0	828.0	13.0	610.0	487.0	11.3	5.3	394.0
BIO65	Vegetable oil fraction (T)	13.0	<0.5	<0.5	8.6	<0.5	1.0	<0.5	<0.5	2.4
BIO84	Safflawer oil (T)	9.6	<0.5	<0.5	<0.5	<0.5	<0.5	<0.5	<0.5	<0.5
BIO85	Linseed oil (T)	12.0	<0.5	2.9	2.1	2.7	<0.5	0.5	<0.5	18.1
BIO130	Tall oil (1) (A) [d]	19.4	<0.5	3.0	6.8	<0.5	76.1	<0.5	<0.5	10.6
BIO131	Tall oil (2) (A) [d]	21.2	<0.5	7.4	10.0	<0.5	156.0	<0.5	<0.5	12.3
BIO132	Tall oil (3) (A) [d]	18.9	<0.5	3.1	8.1	<0.5	81.3	<0.5	<0.5	10.6
BIO145	Tall oil fatty acid (A)	na	<0.5	<0.5	<0.5	<0.5	<0.5	<0.5	<0.5	<1
**Type B**										
BIO19	Palm oil (T)	10.9	2.0	18.6	10.6	5.1	<0.5	<0.5	<0.5	17.6
BIO22	Soybean oil (T)	10.1	<0.5	69.0	19.9	30.1	0.6	1.3	<0.5	85.0
BIO23	Fractioned Seed rape oil (M,D,T)	11.8	<0.5	2.4	5.2	<0.5	125.0	<0.5	<0.5	8.3
BIO38	Corn oil (T)	10.8	<0.5	0.5	<0.5	<0.5	<0.5	<0.5	<0.5	<0.5
BIO54	Animal fat (T)	13.4	<0.5	4.0	27.2	3.3	46.8	0.7	<0.5	90.0
BIO61	Empty fruit bunch (T)	na	0.6	12.6	7.2	1.4	<0.5	0.6	<0.5	9.4
BIO62	Carinata oil (T)	11.0	<0.5	2.8	1.3	0.6	<0.5	<0.5	<0.5	4.6
BIO68	Castor oil (D,T)	13.9	0.6	6.7	2.8	1.5	0.7	<0.5	<0.5	5.2
BIO73	RBD Palm oil (T)	na	na	na	na	na	na	na	na	na
BIO86	Jojoba Oil	5.2	<0.5	4.2	3.4	4.3	<0.5	0.5	<0.5	8.5
BIO87	Cotton Oil (T)	10.3	<0.5	<0.5	<0.5	<0.5	<0.5	<0.5	<0.5	<1
BIO109	Canapa Oil (T)	11.2	<0.5	59.4	140.0	10.8	2.8	<0.5	0.7	65.4

BIO113	Palm kernel oil (T)	23.5	<0.5	1.2	<0.5	<0.5	<0.5	<0.5	<0.5	<1
BIO128	Animal fat (ME)	na	na	na	na	na	na	na	na	na
BIO146	Tobacco oil (T)	9.8	0.6	13.4	52.1	12.9	<0.5	1.4	<0.5	20.6
BIO171	Fatty acids (ME)	na	<0.5	0.8	1.6	<0.5	2.2	<0.5	<0.5	0.9
BIO185	Palm oil mill effluent (A)	12.9	2.4	46.0	28.3	1.7	0.7	0.7	<0.5	20.0
BIO210	Fatty acids (ME)	9.7	<0.5	<0.5	<0.5	<0.5	<0.5	<0.5	<0.5	<1
**TYPE C**										
BIO77	C10SE1 (BE)[e]	12.1	na	na	na	na	na	na	na	na
BIO69	C10SE2 (BE)[e]	na	na	na	na	na	na	na	na	na

Kinetic equations

Autoxidation with radical initiators

Initiation

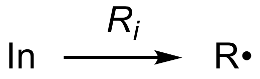

Propagation

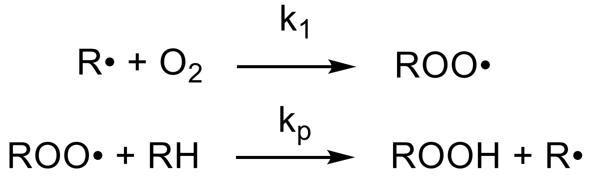

Termination

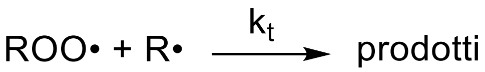

$$-\frac{d[O_2]}{dt} = k_1[R\,\bullet][O_2] \qquad (1)$$

$$\frac{d[R\,\bullet]}{dt} = R_i + k_p[\mathrm{ROO}\,\bullet][\mathrm{RH}] - k_1[R\,\bullet][O_2] \qquad (2)$$

$$\frac{d[\mathrm{ROO}\,\bullet]}{dt} = k_1[R\,\bullet][O_2] - 2k_t[\mathrm{ROO}\,\bullet]^2 - k_p[\mathrm{ROO}\,\bullet][\mathrm{RH}] \qquad (3)$$

Using the steady state approximation for transient species we have:

$$R_i + k_p[\mathrm{ROO}\,\bullet][\mathrm{RH}] - k_1[R\,\bullet][O_2] = 0 \qquad (4)$$

$$k_1[R\,\bullet][O_2] - 2k_t[\mathrm{ROO}\,\bullet]^2 - k_p[\mathrm{ROO}\,\bullet][\mathrm{RH}] = 0 \qquad (5)$$

By combining equations (4) and (5) one obtains:

$$R_i = 2k_t[\mathrm{ROO}\,\bullet]^2 \qquad (6)$$

$$[\mathrm{ROO}\,\bullet] = \sqrt{\frac{R_i}{2k_t}} \tag{7}$$

Substituting equation (7) into (4) we have:

$$k_1[R\,\bullet][O_2] = R_i + k_p\sqrt{\frac{R_i}{2k_t}}[\mathrm{RH}] \tag{8}$$

By replacing (8) with (1) the oxygen consumption rate is obtained:

$$-\frac{d[O_2]}{dt} = \frac{k_p}{\sqrt{2k_t}}[\mathrm{RH}]\sqrt{R_i} + R_i \tag{9}$$

If the chain is then sufficiently long (> 30) it is possible to neglect the oxygen consumption by the inhibitor (Ri) compared to the consumption by the substrate.

Autoxidation inhibited by “chain breaking” antioxidants:

Initiation

$$In \xrightarrow{R_i} R\bullet$$

Propagation

$$R\bullet + O_2 \xrightarrow{k_1} ROO\bullet$$

$$ROO\bullet + RH \xrightarrow{k_p} ROOH + R\bullet$$

Termination

$$ROO\bullet + AH \xrightarrow{k_{in}} A\bullet + ROOH$$

$$ROO\bullet + A\bullet \xrightarrow{k_3} \text{prodotti}$$

$$ROO\bullet + R\bullet \xrightarrow{k_t} \text{prodotti}$$

$$-\frac{d[O_2]}{dt} = k_1[R\bullet][O_2] \qquad (1)$$

$$\frac{d[R\bullet]}{dt} = R_i + k_p[\text{ROO}\bullet][\text{RH}] - k_1[\text{R}\bullet][O_2] \qquad (2)$$

$$\frac{d[\text{ROO}\bullet]}{dt} = k_1[R\bullet][O_2] - 2k_t[\text{ROO}\bullet]^2 - k_p[\text{ROO}\bullet][\text{RH}] + k_{in}[\text{ROO}\bullet][\text{AH}] - k_3[\text{ROO}\bullet][A\bullet] \qquad (3)$$

$$\frac{d[A\bullet]}{dt} = k_{in}[\text{ROO}\bullet][\text{AH}] - k_3[\text{ROO}\bullet][A\bullet] \qquad (4)$$

If applying the steady state approximation, the equations describing the rates of formation of radical species can be set equal to zero, therefore the (2) and the (4) become respectively:

$$k_i[R\bullet][O_2] = R_i + k_p[\text{ROO}\bullet][\text{RH}] \qquad (5)$$

$$k_i[\text{ROO}\bullet][\text{AH}] = k_3[\text{ROO}\bullet][A\bullet] \qquad (6)$$

Substituting these last two expressions in the equation (3) and applying the approximation of the steady state for the ROO• species we have:

$$2k_t[\mathrm{ROO}\,\bullet]^2 + 2k_{in}[\mathrm{ROO}\,\bullet][\mathrm{AH}] - R_i = 0 \qquad (7)$$

By solving the second-degree equation with respect to [ROO•] and ignoring the negative answer, equation 8 is obtained:

$$[\mathrm{ROO}\,\bullet] = \frac{1}{2k_t}\left\{-k_{in}[\mathrm{AH}] + \sqrt{k^2{}_{in}[\mathrm{AH}]^2 + 2k_tR_i}\right\} \qquad (8)$$

Substituting equation (5) in (1) it is obtained:

$$-\frac{d[O_2]}{dt} = k_p[\mathrm{ROO}\,\bullet][\mathrm{RH}] + R_i \qquad (9)$$

Substituting equation (8) for [ROO•] we have:

$$-\frac{d[O_2]}{dt} = \frac{k_p[\mathrm{RH}]}{2k_t}\left\{-k_{in}[\mathrm{AH}] + \sqrt{k^2{}_{in}[\mathrm{AH}]^2 + 2k_tR_i}\right\} + R_i \qquad (10)$$

The expression (10) is general in nature and represents oxygen consumption both in the absence and in the presence of an induction period. In the latter case it is possible to derive a simpler equation: in fact, in the presence of a good antioxidant, the recombination of peroxyls is negligible ($2\,ROO\,\bullet \rightarrow products$) because the termination rate with the antioxidant and the corresponding radical ($ROO\,\bullet$ +AH) are far more important. Based on these considerations, equation (7) becomes:

$$2k_{in}[\mathrm{ROO}\,\bullet][\mathrm{AH}] - R_i = 0 \qquad (11)$$

from which we obtain:

$$[\mathrm{ROO}\,\bullet] = \frac{R_i}{2k_{in}[\mathrm{AH}]} \qquad (12)$$

Substituting equation (12) in (9), the consumption of oxygen in the closed section will be given by:

$$-\frac{d[O_2]}{dt} = \frac{k_p[\mathrm{RH}]R_i}{2k_{in}[\mathrm{AH}]} + R_i \qquad (13)$$

If the chain is long enough, the term R_i can be neglected, and the following equation is obtained:

$$-\frac{d[O_2]}{dt} = \frac{k_p[\text{RH}]R_i}{2k_{in}[\text{AH}]} \quad (14)$$

The formula for determining the *Ri* knowing the induction period is instead obtained from the following equations.

If considering the decay of the inhibitor,

$$-\frac{d[\text{AH}]}{dt} = k_{in}[\text{AH}][\text{ROO} \bullet] \quad (15)$$

in equation (15) we replace (12) and we obtain:

$$-\frac{d[\text{AH}]}{dt} = \frac{R_i}{2} \quad (16)$$

By integrating the differential equation we have:

$$-\int_0^t d[\text{AH}] = \frac{R_i}{2}\int_0^t dt \quad (17)$$

$$[\text{AH}]_0 - [\text{AH}]_t = \frac{R_i}{2}t \quad (18)$$

At time t = τ, what is when all the inhibitor has been consumed, you get:

$$[\text{AH}]_0 = \frac{R_i}{2}\tau \quad (19)$$

Or:

$$R_i = \frac{2[\text{AH}]_0}{\tau} \quad (20)$$

The numerical term that appears in the numerator is the stoichiometric coefficient, that is, the number of peroxyls inactivated by each inhibitor molecule: it is equal to 2 because at the beginning of the calculations it was assumed that all A• radicals trap a peroxyl. With this formula it is therefore possible, from the initiation rate Ri, to obtain the induction time τ or vice versa.
